# Effect of Molecular Flexibility on the Nematic-to-Isotropic Phase Transition for Highly Biaxial Molecular Non-Symmetric Liquid Crystal Dimers

**DOI:** 10.3390/ma4101632

**Published:** 2011-09-27

**Authors:** Nerea Sebastián, David Orencio López, Sergio Diez-Berart, María Rosario de la Fuente, Josep Salud, Miguel Angel Pérez-Jubindo, María Blanca Ros

**Affiliations:** 1 Departamento de Física Aplicada II, Facultad de Ciencia y Tecnología, Universidad del País Vasco, Apartado 644, E-48080 Bilbao, Spain; E-Mails: nerea.sebastian@ehu.es (N.S.); david.orencio.lopez@upc.edu (D.O.L.); rosario.delafuente@ehu.es (M.R.F.); ma.perezjubindo@ehu.es (M.A.P.); 2 Grup de Propietas Físiques dels Materials (GRPFM), Departament de Física i Enginyeria Nuclear, E.T.S.E.I.B., Universitat Politècnica de Catalunya, Diagonal 647, 08028 Barcelona, Spain; E-Mails: sergio.diez@upc.edu (S.D.); josep.salud@upc.edu (J.S.); 3 Departamento de Química Orgánica, Facultad de Ciencias-ICMA, Universidad de Zaragoza, E-50009 Zaragoza, Spain; E-Mail: bros@unizar.es

**Keywords:** liquid crystal dimers, phase transitions, critical exponents, calorimetry, dielectric spectroscopy

## Abstract

In this work, a study of the nematic (N)–isotropic (I) phase transition has been made in a series of odd non-symmetric liquid crystal dimers, the α-(4-cyanobiphenyl-4’-yloxy)-ω-(1-pyrenimine-benzylidene-4’-oxy) alkanes, by means of accurate calorimetric and dielectric measurements. These materials are potential candidates to present the elusive biaxial nematic (N_B_) phase, as they exhibit both molecular biaxiality and flexibility. According to the theory, the uniaxial nematic (N_U_)–isotropic (I) phase transition is first-order in nature, whereas the N_B_–I phase transition is second-order. Thus, a fine analysis of the critical behavior of the N–I phase transition would allow us to determine the presence or not of the biaxial nematic phase and understand how the molecular biaxiality and flexibility of these compounds influences the critical behavior of the N–I phase transition.

## 1. Introduction

Liquid crystals are one kind of molecular materials that, depending on temperature and on concentration in some solvent (lyotropic liquid crystals) or on temperature alone (thermotropic liquid crystals), are able to form intermediate states of matter (mesophases) between the ordered solid crystalline state and the isotropic liquid state. Liquid crystals (also known as mesogens) can be divided in many different sub-types, as they can show a large amount of chemical and physical properties depending, almost completely, on their molecular shape [1].

Conventionally, thermotropic mesogens are formed by a uniaxial rigid core, responsible of the uniaxial arrangement of the molecules in the mesophases, and one or more flexible chains, which act as intermolecular lubricants and, therefore, induce the characteristic fluidity of these materials. The rigid units are usually of two different types, either elongated (calamitic liquid crystals) or disk-like (discotic liquid crystals). In the past decades, there have arisen some new structures for these cores, bent-like shapes being the most active in current research. The most common flexible groups are alkyl chains, although others, as siloxane chains, can also be considered.

Assuming that both uniaxiality as well as flexibility are fundamental properties of thermotropic liquid crystalline molecules, there are different ways of combining both effects. Unlike conventional mesogens, liquid crystal dimers are formed when two rigid cores are linked via a flexible spacer. These materials were first conceived as parts of polymeric structures in order to facilitate their study [2] and have attracted considerable attention during the last years [3,4,5,6,7,8,9,10,11,12,13,14]. When both rigid units (mainly calamitic, but could also be discotic [2] or even bent-like [4,15,16,17]) are identical, the materials are called symmetric liquid crystal dimers, while they are named non-symmetric if the rigid units are different.

On the other side, the nature of the flexible chain has a great and particular influence on molecular shape, which can be summarized in a couple of aspects. In the first place, the chain length, as determined by the number of linking groups, can be viewed as a flexibility-rigidity tuner. The shorter the chain, the more rigid the molecule is, as the mesogenic groups are strongly coupled. Conversely, when the linking chain is long enough, both rigid units can become nearly independent and behave as monomeric liquid crystals, the chain just introducing a slight perturbation in their physical properties. In the second place, the parity of number of carbons in the chain induces what is known as the odd-even effect. Even liquid crystal dimers adopt more anisotropic molecular structures than odd ones, favoring the uniaxial ordering and stabilizing the mesophases at higher temperatures. This translates in higher values for both mesophase-isotropic transition temperatures and entropy changes [3,18,19,20,21,22].

Regardless of the parity of the chain, experimentally verified theoretical models [3,6,11] show that the effect of the flexibility in the molecular shape can be taken into account, mainly, by means of two different molecular conformers (the most probable molecular conformations, indeed) in the whole temperature range; one is more extended and predominates for lower temperatures, while the other one, more isotropic, is preponderant for higher temperatures. The existence of such conformers, and the consequent temperature-dependent-change in the molecular shape, adds still more interest to the study of such compounds, from a fundamental point of view. This could lead to new behaviors (as referent to the monomers) and could induce some of the interesting and desired properties that have been fruitlessly searched during decades. As an example of both properties (flexibility and parity), the bent conformers of calamitic dimers with short chains, could be considered “rigid” liquid crystal dimers or “flexible” bent-like liquid crystals.

One of the most intriguing phenomena in the science of materials is the study of phase transitions, and liquid crystals are, particularly, among the richest systems by far for performing such studies, as they present a great variety of mesophases and phase transitions. The simplest one of these mesophases is the nematic (N) phase, in which the molecules tend to align in the same direction, and this order can be quantified by means of the so called nematic order parameter. Theoretically, the nematic mesophase can be either uniaxial (N_U_) or biaxial (N_B_). The uniaxial nematic phase is characterized by one nematic director, which defines the average direction of the molecules in the mesophase. The biaxial nematic phase needs two directors to be characterized, as the molecules are two-dimensionally aligned. The biaxial nematic phase has been the aim of many experimentalists in the field of liquid crystals. However, its existence in themotropic mesogens has not been undoubtedly determined so far, despite the many hard attempts in this direction [23,24]. Among thermotropic liquid crystals, the potential candidates for presenting the biaxial nematic phase should satisfy some requirements. First of all, the molecules should be biaxial themselves [25,26]. Such molecular biaxiality is one of the main reasons of the current interest on bent molecules, such as V-shaped liquid crystals. Second, as it has been recently claimed, certain flexibility should exist in addition to the biaxial shape of the molecule [27]. It seems that, in order to stabilize the N_B_ phase, a coupling between the orienational order and the conformational distribution would be required. Therefore, liquid crystal dimers appear as excellent candidates to exhibit the elusive biaxial nematic phase, as they fulfill both requirements: molecular biaxiality and molecular flexibility.

One of the ways of identifying a biaxial nematic phase is by means of the nature of the N–I phase transition. As far as the nature of the uniaxial nematic-isotropic (N_U_–I) phase transition is concerned, the Maier-Saupe mean-field approach includes contributions from an attractive potential (derived from an induced dipole moment between adjacent molecules) tending to align the molecular axes, as well as from thermally excited forces which tend to destroy the orientational order [28,29,30,31,32]. The free energy density expansion in powers of the order parameter is considered up to the sixth order and, due to the existence of the cubic invariant term, the N–I phase transition must be first-order in nature. Nevertheless, both molecular biaxiality and flexibility can diminish drastically the value of the cubic invariant term, which could become very small. The fluid-like model [33,34,35] establishes a hypothetical critical region around the transition temperature (*T_NI_*), the limits of which correspond to the spinodal temperatures accounting for the metastable limits of the isotropic and nematic phases, denoted as T* and T**, respectively. In this region, the mean-field approach cannot be applied. If the cubic coeficient becomes zero, both spinodal temperatures should be equal and coincide with the transition temperature, T* = T** = *T_NI_*, which implies that the metastable region (T** − T*) becomes zero.

It had first been theorized that the biaxial nematic-isotropic (N_B_–I) phase transition must be second-order and takes place at the Landau point [36,37], while the N_U_–I phase transition must be first-order [38,39]. Under this assumption, for the biaxial nematic phase to be present, the cubic invariant should be identically zero and the metastable region null. Nevertheless, more recent theoretical studies claim that it is possible to find first-order N_B_–I phase transitions [40,41,42,43], the N_U_–I phase transition being always first-order [44]. In accordance with Allender *et al*. [42], if the biaxial nematic phase is present, there are different possibilities, depending, among other factors, on the “degree of biaxiality” of the mesophase. In states of maximal biaxiality the N_B_–I phase transition is second order and takes place at the Landau point [42], as predicted by the earlier studies [36,37]. When the “degree of biaxiality” is lower, there appears the possibility of finding first-order N_B_–I phase transitions. In such a case, the value of the cubic invariant is smaller for the N_B_–I phase transition than for the N_U_–I phase transition [42]. It is also possible to find, in the same scheme and for adequate values of the cubic invariant, the N_B_–N_U_–I phase sequence, with either a first or a second-order biaxial-uniaxial nematic phase transition [42]. Accurate experimental studies on the nature of the N–I phase transition in liquid crystal dimers may elucidate if this is weakly first or second-order.

For such purposes, in this work three odd members of the non-symmetric liquid crystal dimer series α-(4-cyanobiphenyl-4’-yloxy)-ω-(1-pyrenimine-benzylidene-4’-oxy) alkanes, are considered. From now on, they will be referred as CBOnO.Py, with n being the number of methylene groups in the flexible linking chain. We have chosen odd instead of even members because they seem to adopt more biaxial molecular shapes, as observed from thoeretical models [3,6,11]. The three studied compounds exhibit liquid crystal behavior as reported by Attard *et al*. [45] some time ago, presenting the nematic phase just below the isotropic liquid phase. Our study consists of very accurate calorimetric and static dielectric measurements in order to determine the critical behavior of the materials at the N–I phase transition. Although the compound with n = 11 has been the subject of an extensive study somewhere else [46], it has been considered along with those with n = 7 and 9 with the purpose of an overall comparison.

The structure of the present paper is as follows. In section 2 we describe the experimental details. In section 3 the N–I transition for the studied compounds is analyzed and compared and, finally, the main conclusions are discussed in section 4.

## 2. Experimental Section

### 2.1. Liquid Crystal Materials

The synthesis of CBOnO.Py, with n = 7 and 9, has been reported earlier [45] and the materials have been synthesized accordingly.

**Scheme:**
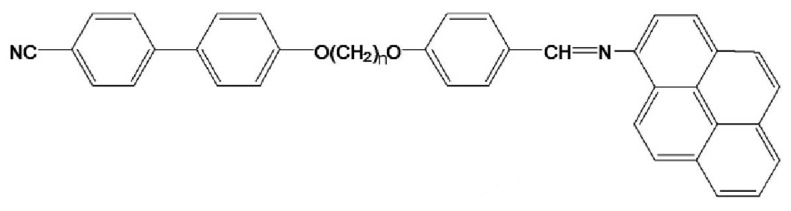
CBOnO.Py molecule.

### 2.2. Specific Heat Measurements

Static specific heat data at constant pressure were obtained through the Modulated Differential Scanning Calorimetry (MDSC) technique via commercial TA instruments Q2000, for which extensive details can be found elsewhere [47,48]. Similar to an AC calorimeter, the MDSC technique, in addition to specific heat data, simultaneously provides phase shift data (*Φ*) that allow us to determine the coexistence region in weakly first-order transitions. The experimental conditions were adjusted in such a way that the phase delay (*ф*) between the modulated heat flow (the response to the perturbation) and the induced temperature oscillations (perturbation) was nearly zero out of the phase transition, and the imaginary part of the complex specific heat data vanished. Similarly, by means of a special calibration procedure in which very precise latent heat data measured from other homologous compounds through adiabatic calorimetry, the MDSC technique is also suitable and allows for quantitative measurements of the latent heat of first order transitions, even when the latent heat is very small.

Typically, measurements were performed in cooling from the I phase down to the mesophase and next on heating; the temperature rate in both cases was 0.01 K min^−1^ with a modulation temperature amplitude (temperature oscillations) of ±0.07 K and a period of 23 s.

### 2.3. Dielectric Measurements

Measurements of the static dielectric permittivity were obtained at a frequency of 10^4^ Hz using the HP 4192A impedance analyzer. The cell consists of two gold-plated brass electrodes (diameter 5 mm) separated by silica spacers, making a plane capacitor of about 50 μm thick. A modified HP 16091A coaxial test fixture was used as the sample holder. It was held in a cryostat made by Novocontrol, and both temperature and dielectric measurements were computer controlled. Additional details of the experimental technique can be found elsewhere [48,49]. Dielectric measurements were performed on cooling at 0.25 K min^−1^.

## 3. Results and Discussion

As mentioned in the introduction, both molecular biaxiality and flexibility are claimed to be necessary conditions to form the themotropic biaxial nematic mesophase, where the cubic invariant could be identically null. In fact, the biaxiality and flexibility of the molecules forming a uniaxial nematic phase diminishes the absolute value of the cubic invariant [39], but it cannot be zero. As a limit case, suggested earlier by Keyes [50] and Anisimov [39], is the N–I tricritical phase transition for which the cubic invariant is very small. In this case, the specific-heat critical exponent *α* on both the N and I phases is 0.5 and, at the same time, the nematic order parameter critical exponent *β* is 0.25.

From an experimental point of view, accurate specific heat and static dielectric data through the N–I phase transition may provide both spinodal temperatures T** and T* or better (T** − T*) which is a measure of the cubic invariant, as well as both *α* and *β* critical exponents.

### 3.1. Specific Heat Measurements

**Figure 1:**
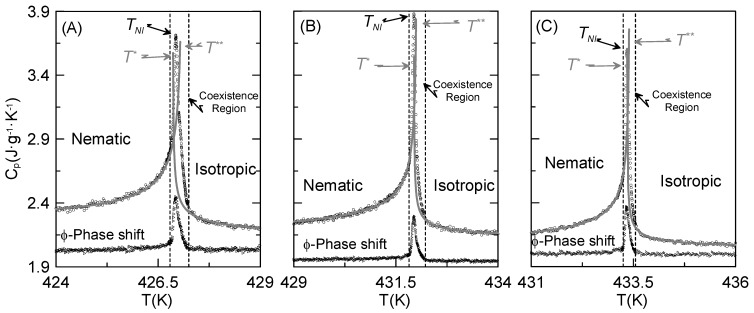
Specific-heat (circles) and *Φ*-phase shift (triangles) data as a function of temperature around the N–I phase transition. The gray lines show the fittings to equations (1a) and (1b). (**A**) CBO11O.Py; (**B**) CBO9O.Py; (**C**) CBO7O.Py.

Specific heat data for the undecane member of the series, CBO11O.Py has been recently determined by us [46]. The behavior of the specific heat in the vicinity of the N–I phase transition is shown in Figure 1A, together with the *ф*-phase shift data, which allows to determine the coexistence region in first-order phase transitions. As it can be observed, the phase transition is discontinuous, as is obvious from the existence of such a phase coexistence region. This result does not allow by itself to determine if the nematic phase of CBO11O.Py is uniaxial or biaxial. However, optical measurements seem to indicate that this the nematic phase is uniaxial [46]. So, the point now is observing whether a change in the flexible spacer length, which will provide a change in both molecular flexibility and biaxiality (as we will prove later), implies a change in the nature of the phase transition and, ultimately, if it is able to drive such a phase transition to second-order.

Figure 1B and Figure 1C show both the specific-heat and *ф*-phase shift data for the next two odd-members of the series with shorter chains: CBO9O.Py and CBO7O.Py, respectively. It can also be seen how in both compounds the N–I phase transition is first-order in nature, as it has been found for the CBO11O.Py. It should be stressed that no signature of a possible N_B_–N_U_ phase transition is observed for any of the three compounds that have been calorimetrically studied down to more than 30 degrees under the N–I phase transition [46]. Nevertheless, Cordoyiannis *et al*. [51] did not observe, by means of adiabatic scanning calorimetry, a reported N_B_–N_U_ phase transition [52].

Transition temperatures, *T_NI_*, for the three compounds, are listed in Table 1 (obtained by us in this work and ref. in [46]), along with those reported by Attard *et al*. [45]. To study how the molecular flexibility influences the first-order N–I phase transition, two actions in our analysis will be taken into account. The first one consists in determining the width of the metastable region (T** − T*) and the second one, in obtaining accurately the latent heat. 

**Table 1:** Nematic-isotropic transition temperatures and enthalpy changes.

CBOnO.Py	T_N–I_ (K)	ΔH_N–I_ (kJ·mol^−1^)	Ref.
CBO11O.Py	426.9 432.2	0.5 ± 0.1 1.8	[46] [45]
CBO9O.Py	431.95 434.15	0.33 ± 0.07 1.2	This work [45]
CBO7O.Py	433.35 437.15	0.32 ± 0.08 1.0	This work [45]

To obtain both spinodal temperatures (T* and T**) at the N–I phase transition, the specific-heat data will be fitted to the standard expressions [39,46,49]:

(1a) $$C_{p,I}=B_{C}+D_{C}\left[\frac{T}{T^{*}}-1\right]+A_{C,I}{\left|\frac{T}{T^{*}}-1\right|}^{-\alpha }\;\;\;for\;\;\;T>T_{NI}=T^{*}+\Delta T^{*}$$

(1b) $$C_{p,N}=B_{C}+D_{C}\left[\frac{T}{T^{**}}-1\right]+A_{C,N}{\left|\frac{T}{T^{**}}-1\right|}^{-\alpha }\;\;\;for\;\;\;T<T_{NI}=T^{**}-\Delta T^{**}$$

where *α* is the specific heat critical exponent, the same at the I and N phases. Both *B_C_* and *D_C_* terms concern to the so called specific heat background, being identical at both sides of the transition. The last term in both equations is the critical power law divergence, where *A_C,N_* and *A_C,I_* are the corresponding amplitudes. Common parameters in both phases (*B_C_*, *D_C_* and α) have been simultaneously refined after a previous independent fitting.

The most significant parameters (*A_C,N_/A_C,I_*; (T^**^ − T^**^) and *α*) that characterize the nature of the N–I phase transition for the three compounds are collected in Table 2. All the fitted parameters represent well enough the measured specific heat data, as indicated by χ^2^ values.

**Table 2:** Fitting parameters from the specific-heat and dielectric measurements.

	*α*	T** − T* (K)	*A_N_/A_I_*	χ^2^ × 10^4^	Measurement
CBO11O.Py	0.51 ± 0.05 0.51 ± 0.05	0.21 0.42	2.7 ± 0.1 −0.90 ± 0.02	2 0.4	Specific-heat Dielectrics
CBO9O.Py	0.50 ± 0.02 0.5 ± 0.1	0.08 0.24	3.1 ± 0.1 −2.5 ± 0.1	1 1	Specific-heat Dielectrics
CBO7O.Py	0.50 ± 0.01 0.50 ± 0.01	0.07 1.53	2.8 ± 0.1 −1.70 ± 0.01	0.7 0.05	Specific-heat Dielectrics

**Figure 2:**
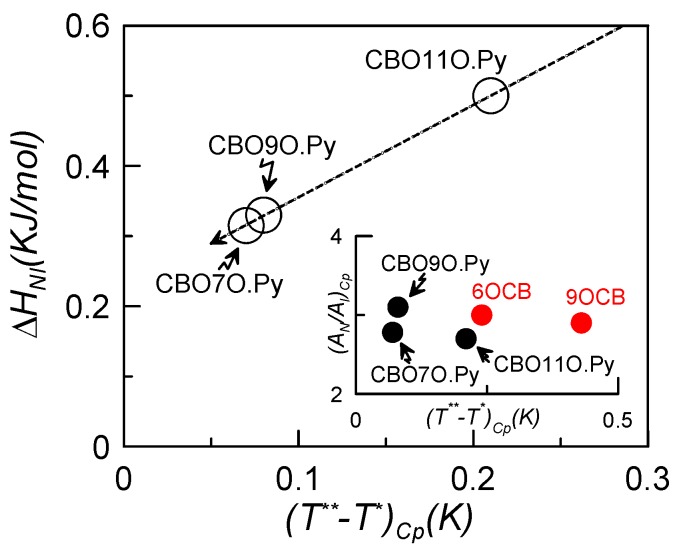
Latent heat at the N–I phase transition *vs.* the metastable region for the three studied dimers. The inset shows the ratio between the nematic and isotropic amplitudes of the critical power law divergence (*A_C,N_/A_C,I_*) in a function of the metastable region for the studied dimers and two liquid crystal monomers.

As it can be seen from Table 2, it seems that the shorter the flexible linking chain of the dimer, the narrower the metastable region (T** − T*) is. Even if all of them have small values, indicating that they all present very weak first-order N–I phase transitions, Table 2 shows that the metastable region corresponding to the undecane member (n = 11) (obtained from calorimetric measurements) is about 2–3 times larger than those corresponding to the two other members, which exhibit extremely weak first-order transitions. The *A_C,N_/A_C,I_* ratio *vs*. the metastable region is plotted in the inset of Figure 2 for the three studied compounds along with two liquid crystal monomers for which the tricritical N–I phase transition was reported [44,49]. The values of the critical exponent *α*, as shown in Table 2, are of about 0.5, in accordance with the tricritical hypothesis [39], as well. It is evidenced that the *A_C,N_/A_C,I_* ratio is about 3, the value usually found for a tricritical behavior.

The total enthalpy change associated to the N–I phase transition ($\Delta H_{NI}^{TOT}$) can be written as:

(2) $$\Delta H_{NI}^{TOT}=\Delta H_{NI}+\int \Delta C_{p}dT$$

where Δ*H_NI_* is the latent heat and the second term of the right-hand is the pretransitional fluctuation contribution (Δ*C_p_* is calculated as *C_p_-C_p,background_* through *B_C_* and *D_C_* parameters in Equations (1a) and (1b). The latent heat can be individually obtained from equation (2) by performing a careful calibration [49].

Our values of latent heat, along with those read from the literature are also consigned in Table 1. As it can be clearly observed from such a table, our calculated latent heat is about three and a half times lower than the value reported by Attard *et al*. [45]. In order to make a more complete analysis, we compare the latent heat and the metastable region in Figure 2 for the three investigated liquid crystal dimers. These results confirm those obtained for the metastable regions, as the latent heat is larger for the undecane dimer, and shorter for the heptane (n = 7), as the value for the nonane (n = 9) lays in between the other two. This is, the shorter the chain, the weaker the first-order phase transition is.

As a consequence of these results, we would like to stress the following point. It has been mentioned above that both molecular biaxiality and flexibility must be instrinsic characteristics that could potentially lead to the formation of a biaxial nematic phase [27,28,29]. The fact that the cubic invariant diminishes as the spacer is shortened, making the first-order phase transition weaker, seems to indicate that the nematic phase is “closer” to biaxial or, even, biaxial. The presented results claim that there is a competition between these two features in the studied compounds. It could appear that the most flexible dimer, the CBO11O.Py, should be closer to present the biaxial nematic phase and, thus, to present the weakest first-order phase transition among the three members. This is clearly not the case. 

The possible explanation could be in the fact that, as the chain is shorter, the molecules on average are more biaxial. As it has been mentioned before, there are two main molecular conformations for these compounds. In the case of odd dimers, we can find an extended (*trans*) conformation, in which the angle between the two rigid cores is about 109° and a hairpin (*cis*) conformation with an angle of 0° between both cores (the cores are parallel to each other). The *trans* conformation is more anisotropic than the *cis* one (more isotropic) and, therefore, the trans conformation is more biaxial, as well. In the whole temperature range, the *trans* conformation is dominant for the three studied compounds, but its population (relative to the *cis* one) varies with the length of the spacer. So, as long as the *trans* population is higher, there are more biaxial molecules and the “overall molecular biaxiality” is higher. The *trans* and *cis* populations in the whole temperature range of the three studied dimers can be estimated from the Stocchero’s model [6] applied to dielectric relaxation data. The estimated values for the trans population in the N mesophase close to the N–I transition are 76% for the CBO11O.Py [46], 79% for the CBO9O.Py and 82% for the CBO7O.Py [53]. This confirms the fact that the overall molecular biaxiality is higher for the shortest homologue (n = 7) and lower for the longest one (n = 11), with the one in the middle (n = 9) having a *trans* population (and an overall molecular biaxiality) in between the other two.

The question that now arises is: is the difference in the populations of the conformers enough to induce such changes in the nature of the N–I phase transition? There may be some other causes that help the molecular biaxiality to increase as the length of the spacer decreases, such as, for example, a slight variation in the angle between the two rigid cores, with shorter homologues having smallest angles (in the *trans* conformation) and the molecules being even more biaxial. Anyway, the ultimate point is that, on average, the longer the chain the “less” biaxial the molecular shape and, as results uphold, the molecular biaxiality has a greater influence than the flexibility in “driving” the uniaxial nematic mesophase to biaxial.

It is not possible to dilucidate, just with these results, if the nematic mesophase is uniaxial or biaxial, for the shortest homologues: CBO9O.Py and CBO7O.Py.

With these considerations in mind, and even if the biaxial mesophase is not present in these compounds, it could be expected from what is observed in Figure 2 that the biaxial nematic phase would indeed appear for shorter odd homologues of the same series (this is, CBO5O.Py and CBO3O.Py).

### 3.2. Dielectric Measurements

**Figure 3:**
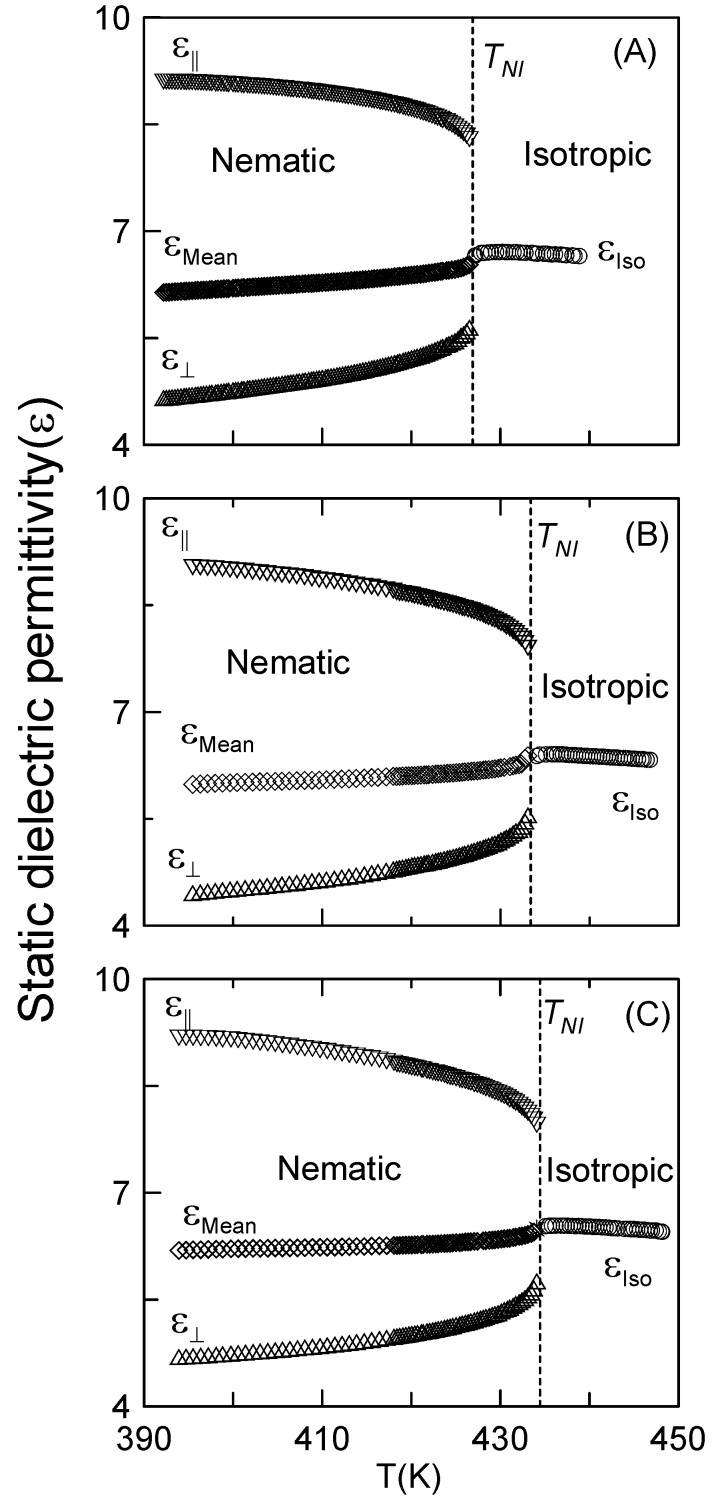
Static dielectric permittivity *vs*. temperature for (**A**) CBO11O.Py; (**B**) CBO9O.Py; (**C**) CBO7O.Py. In the nematic phase, downward triangles represent the parallel component of the static dielectric permittivity, upward triangles represent the perpendicular component and diamonds represent the mean value. Circles represent the static dielectric permittivity in the isotropic phase.

**Figure 4:**
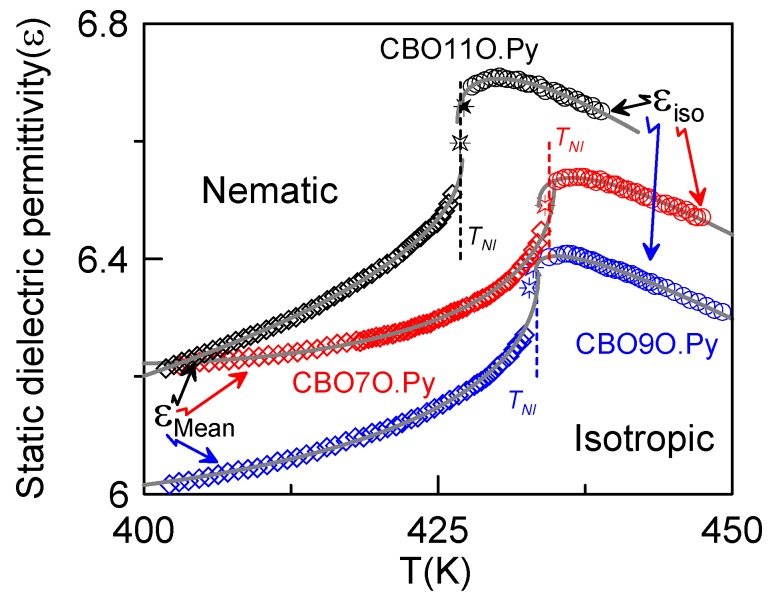
Behavior of the mean static dielectric permittivity (diamonds) in the nematic phase and the static dielectric permittivity (circles) in the isotropic phase for the three liquid crystal dimers. Gray solid lines are fittings according to equations (4a) and (4b).

We recently undertook the analysis of the static dielectric permittivity and its critical behavior in the CBO11O.Py [46]. The static permittivity of the CBO11O.Py in function of the temperature is shown in Figure 3A for the nematic phase (for both parallel and perpendicular components and the mean value) and the isotropic phase. Figure 3B and Figure 3C show the static permittivity *vs*. temperature for the CBO9O.Py and CBO7O.Py dimers, respectively. Details of the evolution of the dielectric permittivity data (*ε_Mean_* and *ε_iso_*) around the N–I phase transitions are shown in Figure 4, where the data analysis has been carried out according to the following equations [49,54].

(4a) $$\varepsilon_{iso}=\varepsilon^{*}+a_{I}\left|T-T^{*}\right|+A_{\varepsilon ,I}{\left|T-T^{*}\right|}^{1-\alpha }\;\;\;for\;\;\;T>T_{NI}=T^{*}+\Delta T^{*}$$

(4b) $$\varepsilon_{mean}=\varepsilon^{**}+a_{N}\left|T-T^{**}\right|+A_{\varepsilon ,N}{\left|T-T^{**}\right|}^{1-\alpha }\;\;\;for\;\;\;T<T_{NI}=T^{**}-\Delta T^{**}$$

where *α* is the specific heat critical exponent, T^*^, T^**^, ΔT^*^ and ΔT^**^ have the same meaning as in equations (1). The parameters ε^*^ and ε^**^ are, respectively, the extrapolated values of *ε_iso_* and *ε_mean_* at T^*^, T^**^. Both *a_I_* and *a_N_* are the static dielectric permittivity background terms and *A_ε,I_* and *A_ε,N_* are the corresponding dielectric amplitudes. It has to be noticed that the data corresponding to the star symbols in Figure 4 have been removed from the fitting, according to the previously used procedure [46]. The corresponding fittings for the three studied dimers proved to be extremely good, as can be observed from Figure 4. The value of the critical exponent for the three compounds is in accordance with the tricritical hypothesis (*α* = 0.5), as has also been the case for the fittings of the specific-heat data. The values of the fitting parameters are consigned in Table 2.

In order to ultimately confirm or discard the tricritical nature of the N–I phase transition in the investigated dimers, the nematic order parameter critical exponent should be obtained. In liquid crystal monomers, the dielectric anisotropy is usually taken as proportional to the nematic order parameter. The case of CBOnO.Py dimers seems more complex. However, as long as the *trans* conformers’ population is significantly much higher than that of the *cis* conformers, the resulting picture is that the dimer seems to be formed by pseudo-elongated molecules (*trans* conformers) with a longitudinal dipole (the transverse component is negligible) moment as in the case of monomers. Furthermore, it can be observed in Figure 3 how well the static dielectric permittivity behavior with temperature looks like that of a typical monomer with a nearly longitudinal dipole moment. As a consequence, it should be reasonable to consider the dielectric anisotropy of these compounds as proportional to the nematic order parameter as well.

**Figure 5:**
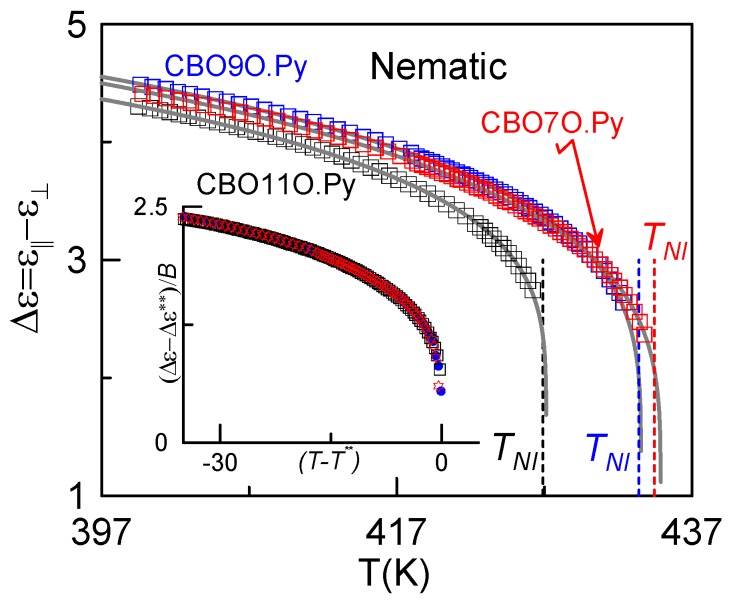
Dielectric anisotropy as a function of temperature for the three studied dimers. Gray solid lines are fittings according to Equation (5). The inset shows the dependence of $\left(\Delta \varepsilon -\Delta \varepsilon^{**}\right)/\;B$ with the metastable region for CBO11O.Py, CBO9O.Py and CBO7O.Py.

The critical behavior of *Δε* at the N-to-I phase transition is usually parameterized, according to the Landau-de Gennes theory [39,54] as

(5) $$\Delta \varepsilon =\Delta \varepsilon^{**}+B{\left|T-T^{**}\right|}^{\beta }\;\;\;for\;T<T^{**}-\Delta T^{**}$$

where T^**^ has the same meaning as in eqs. (1b) and (4b), and *β* is the critical exponent of the nematic order parameter. Figure 5 shows the dielectric anisotropy data as a function of temperature in the nematic phase for the three studied dimers. In the inset of Figure 5 it can be observed that the critical behavior of the three dimers is identical at the N–I phase transition. Finally, as shown in Table 2, the fitting parameters also confirm the tricritical character of this transition for the three compunds, as the value of the critical exponent (*β* = 0.25) (the same for all of them) is that predicted by the tricritical hypothesis.

## 4. Conclusions

The results of the critical behavior of the N–I phase transition of the three homologues odd asymmetric liquid crystal dimers CBOnO.Py with n = 11, 9 and 7 have been summarized. The critical behavior was analyzed both by means of calorimetric (specific-heat) and dielectric (static dielectric permittivity as well as dielectric anisotropy) data.

The possibility of finding the interesting biaxial nematic phase was the departure point of this work, and these materials, presenting both molecular biaxiality and flexibility were regarded as good candidates for such a purpose. In all cases (for the three compounds and by all the techniques), however, the N–I phase transitions were proved to be first-order in nature, following the tricritical hypothesis. As a conclusion, we are not able to determine if the nematic mesophase of CBO9O.Py and CBO7O.Py are uniaxial or biaxial with these results alone. With respect to the possibility of finding a N_B_-N_U_-I phase sequence, we did not observe any indication of a biaxial-uniaxial nematic phase transition neither by calorimetry nor by dielectrics.

With a further analysis of the calorimetric results, by means of the width of the metastable region and the value of the transitional enthalpy change, it could be stated how the more rigid the molecule (this is, the smaller the value of n), the weaker the first-order phase transition is. These results could be explained by the simultaneous contribution of several different causes that leads to a higher level of the overall molecular biaxiality as the chain gets shorter. Such an increase in the molecular biaxiality overcomes the decrease in the molecular flexibility, the first-order N–I phase transition becoming weaker while the chain gets shorter. It seems to be confirmed that the *trans* conformers population is higher as long as the flexible spacer is shorter. Another possibility to be confirmed is that the relative angle between the two rigid cores of the liquid crystal dimer (in the *trans* conformation) is probably different for the three studied compounds. It seems that the “overall” molecular biaxiality is more pronounced for the shorter homologues.

The final questions are: do the CBO9O.Py and CBO7O.Py compounds present the biaxial nematic mesophase? And if they do not, could such a mesophase exist in the shorter odd-members of the CBOnO.Py series? It is evident that further experiments are needed to confirm or discard the existence of the biaxial nematic phase in this kind of compounds.

## References

[1] Demus D., Goodby J.W., Gray G.W., Spiess H.W., Vill V. (1998). Handbook of Liquid Crystals Vol. 1: Fundamentals.

[2] Imrie C.T., Luckhurst G.R., Demus D., Goodby J.W., Gray G.W., Spiess H.W., Vill V. (1998). Liquid crystal dimers, oligomers. Handbook of Liquid Crystals Vol. 2B: Low Molecular Weight Liquid Crystals.

[3] Ferrarini A., Luckhurst G.R., Nordio P.L., Roskilly S.J. (1993). Understanding the unusual transitional behaviour of liquid crystal dimmers. Chem. Phys. Lett..

[4] Tamba M.G., Kosata B., Pelz K., Diele S., Pelzl G., Vakhovskaya Z., Kresse H., Weissflog W. (2006). Mesogenic dimers composed of a calamitic and a bent-core mesogenic unit. Soft Matte..

[5] Dunmur D.A., Luckhurst G.R., de la Fuente M.R., Diez S., Pérez-Jubindo M.A. (2001). Dielectric relaxation in liquid crystalline dimmers. J. Chem. Phys..

[6] Stocchero M., Ferrarini A., Moro G.J., Dunmur D.A., Luckhurst G.R. (2004). Molecular theory of dielectric relaxation in nematic dimmers. J. Chem. Phys..

[7] Coles H.J., Pivnenko M.N. (2005). Liquid crystal ‘blue phases’ with a wide temperature range. Nature.

[8] Takanishi Y., Toshimitsu M., Nakata M., Takada N., Izumi T., Ishikawa K., Takezoe H., Watanabe J., Takahasi Y., Iida A. (2006). Frustrated smectic layer structuresin bent-shaped dimer liquid crystalsas studied by X-ray microbeam diffraction. Phys. Rev. E.

[9] Imrie C.T., Henderson P.A. (2007). Liquid crystal dimers and higher oligomers: Between monomers and Polymers. Chem. Soc. Rev..

[10] Sepelj M.S., Baumeister U., Diele S., Nguyen H.L., Bruce D.W. (2007). Intercalated liquid-crystalline phases formed by symmetric dimers with an α,ω-diiminoalkylene spacer. J. Mat. Chem..

[11] Ferrarini A., Greco C., Luckhurst G.R. (2007). On the flexoelectric coefficients of liquid crystal monomers and dimers: A computational methodology bridging length-scales. J. Mater. Chem..

[12] Aziz N., Kelly S.M., Duffy W., Goulding M. (2008). Banana-shaped dopants for flexoelectric nematic mixtures. Liq. Cryst..

[13] de la Fuente M.R., López D.O., Pérez-Jubindo M.A., Dunmur D.A., Diez-Berart S., Salud J. (2010). Cylindrical sub-micrometer confinement results for the odd-symmetric dimer α,ω-bis[(4-cyanobiphenyl)-4’-yloxy]undecane (BCB.O11). J. Phys. Chem. B.

[14] Cestari M., Diez-Berart S., Dunmur D.A., Ferrarini A., de la Fuente M.R., Jackson D.A., López D.O., Luckhurst G.R., Pérez-Jubindo M.A., Richardson R.M., Salud J., Timini B.A., Zimmermann H. (2011). The phase behaviour and properties of the liquid crystal dimer α,ω-bis[(4-cyanobiphenyl)-4’-yl]-heptane: A novel twist-bend nematic?. Phys. Rev. E.

[15] Lagerwall J.P.F., Giesselmann F., Wand M.D., Walba D.M. (2004). A chameleon chiral polar liquid crystal: Rod-shaped when nematic, bent-shaped when smectic. Chem. Mater..

[16] Umadevi S., Sadashiva B.K., Murthy H.N., Raghunathan V.A. (2006). Mesogenic dimers composed of bent-core molecules with flexible alkylene spacer. Soft Matter.

[17] Umadevi S., Sadashiva B.K. (2007). Liquid crystal properties and dependence of transition temperatures on the length of the flexible alkylene spacer of symmetric dimers composed of bent-core units. Liq. Cryst..

[18] Marcelja S. (1974). Chain ordering in liquid crystals. I. Even-odd effect. J. Chem. Phys..

[19] Emsley J.W., Luckhurst G.R., Shilstone G.N. (1984). The orientational order of nematogenic molecules with a flexible core. A dramatic odd even effect. Mol. Phys..

[20] Ferrarini A., Luckhurst G.R., Nordio P.L., Roskilly S.J. (1994). Prediction of the transitional properties of liquid crystal dimers. A molecular-field calculation based on the surface tensor parametrization. J. Chem. Phys..

[21] Luckhurst G.R. (1994). The Marcelja-Luckhurst molecular-field for uniaxial nematics composed of flexible molecules. A variational derivation. Mol. Phys..

[22] Luckhurst G.R., Romano S. (1997). Computer simulation studies of anisotropic systems. 26. Liquid crystal dimers: A generic model. J. Chem. Phys..

[23] Berardi R., Zannoni C. (2000). Do thermotropic biaxial nematics exist? A montecarlo study of biaxial Gay-Berne particles. J. Chem. Phys..

[24] Luckhurst G.R. (2001). Biaxial nematic liquid crystals: Fact or a fiction?. Thin Solid Films.

[25] Galerne Y. (2006). Comment on “Thermotropic biaxial nematic liquid crystals”. Phys. Rev. Lett..

[26] Madsen L.A., Dingemans T.J., Nakata M., Samulsky E.T. (2006). Comment on “Thermotropic biaxial nematic liquid crystals”. Phys. Rev. Lett..

[27] Luckhurst G.R. (2004). Liquid crystals. A missing phase found at last?. Nature.

[28] Luckhurst G.R. (2005). V-shaped molecules: New contenders for the biaxial nematic phase. Angew. Chem. Int. Ed..

[29] Luckhurst G.R. (2009). Biaxial nematics composed of flexible molecules: A molecular field theory. Liq. Cryst..

[30] Maier W., Saupe A. (1958). Eine einfache molekulare theorie des nematischen kristallinflussigen zustandes. Z. Naturforsch..

[31] Maier W., Saupe A.Z. (1959). Eine einfache molekular-statistische theorie der nematischen kristallinflussigen phase.1. Naturforsch.

[32] Maier W., Saupe A.Z. (1960). Eine einfache molekular-statistische theorie der nematischen kristallinflussigen phase. 2. Naturforsch.

[33] Mukherjee P.K., Mukherjee T.B. (1995). Critical region of the nematic-isotropic phase-transition in the epsilon expansion. Phys. Rev. B.

[34] Wang Z.H., Keyes P.H. (1996). Critical and multicritical fluctuations of nematic liquid crystals. Phys. Rev. E.

[35] Mukherjee P.K. (1998). The T_N–I_ − T* puzzle of the nematic-isotropic phase transition. J. Phys..

[36] Freiser M.J. (1970). Ordered states of a nematic liquid. Phys. Rev. Lett..

[37] Boccara N., Mejdani R., de Seze L. (1977). Solvable model exhibiting a 1st order phase-transition. J. Phys..

[38] De Gennes P.G. (1994). The Physics of Liquid Crystals.

[39] Anisimov M.A. (1991). Critical Phenomena in Liquids and Liquid Crystals.

[40] De Matteis G., Virga E.G. (2005). Tricritical points in biaxial liquid crystal phases. Phys. Rev. E.

[41] Bisi F., Romano S., Virga E.V. (2007). Uniaxial rebound at the nematic biaxial transition. Phys. Rev. E.

[42] Allender D., Longa L. (2008). Landau-de Gennes theory of biaxial nematics re-examined. Phys. Rev. E.

[43] Tschierske C., Photinos D.J. (2010). Biaxial nematic phases. J. Mat. Chem..

[44] Salud J., Cusmin P., de la Fuente M.R., Pérez-Jubindo M.A., López D.O., Diez-Berart S. (2009). Study of the critical behavior and scaling relationships at the N–to–I phase transition in hexyloxycyanobiphenyl. J. Phys. Chem. B.

[45] Attard G.S., Imrie C.T. (1992). Low molar mass liquid-crystalline glasses: Preparation and properties of the α-(4-Cyanobiphenyl-4’-oxy-) ω-(1 -pyreniminebenzylidene-4’-oxy)alkanes. Chem. Mater..

[46] Sebastián N., de la Fuente M.R., López D.O., Pérez-Jubindo M.A., Salud J., Diez-Berart S., Ros M.B. (2011). Dielectric and thermodynamic study of the liquid cristal dimer α-(4-cyanobiphenyl-4’-oxy)-ω-(1-pyreniminebenzylidene-4’-oxy) undecane (CBO11O.Py). J. Phys. Chem. B.

[47] Sied M.B., Salud J., López D.O., Barrio M., Tamarit J.L. (2002). Binary mixtures of nCB and nOCB liquid crystals. Two experimental evidences for a smectic A-nematic tricritical point. Phys. Chem. Chem. Phys..

[48] Puertas R., Rute M.A., Salud J., López D.O., Diez S., Van Miltenburg J.C., Pardo L.C., Tamarit J.L., Barrio M., Pérez-Jubindo M.A., de la Fuente M.R. (2004). Thermodynamic, crystallographic, and dielectric study of the nature of glass transitions in cyclo-octanol. Phys. Rev. B.

[49] Cusmin P., de la Fuente M.R., Salud J., Pérez-Jubindo M.A., Diez-Berart S., López D.O. (2007). Critical behavior and scaling relationships at the SmA_d_–N and N–I transitions in nonyloxycyanobiphenyl (9OCB). J. Phys. Chem. B.

[50] Keyes P.H., Shane J.R. (1979). Tricritical exponents for the isotropic-nematic transition: Experimental-verification. Phys. Rev. Lett..

[51] Cordoyiannis G., Apreutesei D., Mehl G.H., Glorieux C., Thoen J. (2008). High-resolution calorimetry study of a liquid crystalline organo-siloxane tetrapode with a biaxial nematic phase. Phys. Rev. E.

[52] Merkel K., Kocot A., Vij J.K., Korlacki R., Mehl G.H., Meyer T. (2004). Thermotropic biaxial nematic phase in liquid crystalline organo-siloxane tetrapodes. Phys. Rev. Lett..

[53] Sebastián N., de la Fuente M.R., López D.O., Pérez-Jubindo M.A., Salud J., Diez-Berart S., Ros M.B. (2011). Dielectric studies of the odd liquid cristal dimers α-(4-cyanobiphenyl-4’-oxy)-ω-(1-pyreniminebenzylidene-4’-oxy) alkanes (CBOnO.Py).

[54] Rzoska S.J., Ziolo J., Sulkowski W., Jadzyn J., Czechowski G. (2001). Fluidlike behavior of dielectric permittivity in a wide range of temperature above and below the nematic-isotropic transition. Phys. Rev. E.

